# Risk Factors for Severe Erectile Dysfunction after Focal Therapy with High-Intensity Focused Ultrasound for Prostate Cancer

**DOI:** 10.3390/biomedicines10112876

**Published:** 2022-11-10

**Authors:** Sunao Shoji, Satoshi Kuroda, Kohei Uemura, Kazuya Oda, Tatsuo Kano, Takahiro Ogawa, Tatsuya Umemoto, Mayura Nakano, Masayoshi Kawakami, Masahiro Nitta, Masanori Hasegawa, Akira Miyajima

**Affiliations:** 1 Department of Urology, Tokai University School of Medicine, 143 Shimokasuya, Isehara 259-1143, Japan; 2 Biostatistics and Bioinformatics Course, The University of Tokyo, 7-3-1 Hongo, Bunkyo-ku, Tokyo 113-8654, Japan

**Keywords:** sexual function, localized prostate cancer, focal therapy, high-intensity focused ultrasound, treated area

## Abstract

The present study aimed to analyze the effect of predisposing clinical factors for severe erectile dysfunction (ED) in patients treated with focal therapy using high-intensity focused ultrasound (HIFU) for localized prostate cancer (PC). Patients without severe ED (International Index of Erectile Function-5 [IIEF-5] score ≥ 8) before focal HIFU therapy were included. A total of 92 of the 240 patients met the inclusion criteria and were included. The rate of severe ED (IIEF-5 ≤ 7) was 36% 12 months after treatment. Multivariable logistic regression analysis showed that the pre-procedural lower IIEF-5 score (odds ratio [OR] 0.812, *p* = 0.005), the pre-procedural lower score of the sexual domain of the Expanded Prostate Cancer Index Composite (OR 0.960, *p* = 0.038), and the treatment of the edge of the peripheral zone (PZ) in proximity to the neurovascular bundle (NVB) [treated vs. untreated, OR 8.048, *p* = 0.028] were significant risk factors for severe ED at 12 months after treatment. In conclusion, pre-procedural lower erectile function and treatment of the part in proximity to the NVB were significant risk factors for severe ED after focal therapy.

## 1. Introduction

Treatment of localized prostate cancer (PC) can lead to sexual dysfunction, such as erectile dysfunction (ED), significantly affecting the quality of life (QOL) of men after treatment. The neurovascular bundle (NVB) has previously been reported as a structure relevant for erectile function [1]. Although focal therapy is an investigational treatment that can cure clinically significant PC (csPC) while preserving the anatomical structures related to sexual function, such as the NVB and internal pudendal arteries [2], rates of severe ED have been observed in 10–50% of patients undergoing focal therapy [3]. In a previous comparison of focal therapy (n = 195) and whole-gland therapy (n = 105) with high-intensity focused ultrasound (HIFU) or cryotherapy, the median International Index of Erectile Function-5 (IIEF-5) scores for focal therapy and whole-gland therapy were 12 and 5 (*p* < 0.001), respectively, at 3 months. The median IIEF-5 scores were 13 and 9 (*p* = 0.04) at 12 months after treatment. However, there was no significant difference between focal therapy and whole-gland therapy based on median IIEF-5 scores (15 vs. 14, *p* = 0.4) prior to treatment [4]. 

HIFU is an extracorporeal ablative technology that delivers ultrasonic energy to pinpoint foci that are only millimeters wide, and only minor temperature changes are observed outside of the focal zone [5], making it an attractive modality for focal therapy [6,7]. However, as a feature of HIFU, heat dissemination to the surrounding cavernous nerve and pudendal arteries in up to 75% of men who developed severe ED was observed after whole-gland HIFU [8]. Therefore, the negative influence of HIFU would be hypothesized for erectile function after the treatment of the treatment area close to the NVB, such as the edge of the peripheral zone (PZ) in proximity to the NVB. 

Because ED was reported after focal therapy with HIFU, the analysis of the risk of ED after treatment would contribute to the informed consent of patients who are interested in treatment. The present study aimed to analyze the effect of clinical factors predisposing to severe ED in patients treated with focal therapy using HIFU for localized prostate cancer (PC). A clinical factor was whether the treatment area included the edge of the PZ and the adjacent NVB.

## 2. Materials and Methods

### 2.1. Patients

Pooled functional data from prospective, registered, institutional reviewer board-approved (21R038), and externally audited studies conducted in Japan between 2016 and 2020 (Japan Registry of Clinical Trials, jRCTs032180303) were used for the analysis. Focal therapy was performed in patients with serum prostate-specific antigen (PSA) levels ≤ 20 ng/mL, and csPC was detected using magnetic resonance imaging (MRI)-transrectal ultrasound (TRUS) fusion image-guided transperineal prostate biopsy with BioJet^®^ (D&K Technologies GmbH, Barum, Germany) and 12-core transperineal template-assisted systematic biopsy. The patients were informed that focal therapy was optional in addition to the standard treatment, and those who underwent the procedure provided informed consent. We prospectively included patients with moderate (IIEF-5 score 8–11), moderate-to-mild (IIEF-5 score 12–16), mild (IIEF-5 score 17–21), or no ED (IIEF-5 score 22–25) [9] without a phosphodiesterase type 5 inhibitor (PDE-5 inhibitor) or other treatment for ED before treatment. Patients with abnormal renal function were excluded from this study because dynamic contrast-enhanced (DCE) MRI was used to evaluate the effect of the treatment on blood flow in the prostate. Furthermore, we excluded patients with diseases or habits that could affect sexual function, such as diabetes mellitus, coronary artery disease, neurological disorders, obesity, or current smoking habits. 

### 2.2. Treatment Protocol

The detailed protocol for focal therapy with HIFU has been previously described [6]. csPC localization was treated with HIFU using Sonablate^®^ 500 (SonaCare Medical, Indianapolis, IN, USA). Sonablate^®^ 500 uses proprietary transducer technology with low-energy ultrasound (4 MHz) to image the prostate and high-energy ablative pulses (site intensity:1300–2200 W/cm^2^) to deliver treatment. Intraoperative ultrasound images were obtained during the treatment. Based on the appearance of the popcorn phenomenon in the target area, which indicates effective treatment, energy output can be adjusted intraoperatively from 24 W to 48 W [6]. Within 24 h after treatment, the urethral catheter was removed, and the patients were discharged. The absence of blood flow in the treated area 2–4 weeks after treatment on DCE-MRI was considered a successful treatment. After the procedure, no further treatment for sexual function such as a PDE-5 inhibitor was administered.

### 2.3. Evaluation of Sexual Function after the Treatment

**Figure 1:**
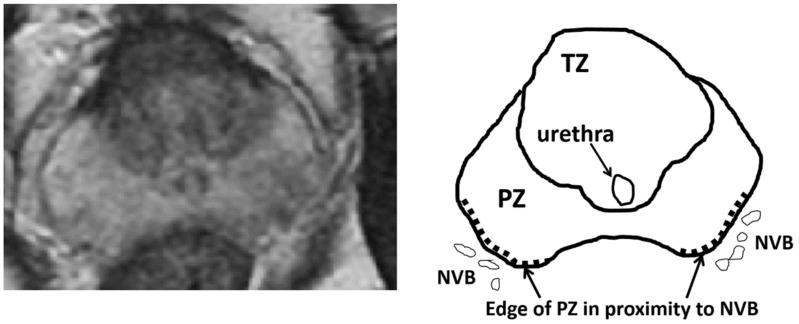
Therapeutic area of the peripheral zone with an adjacent neuro vascular bundle. The figures show axial MRI images of the prostate and the therapeutic areas of the peripheral zone with adjacent neurovascular bundles (dotted line). Abbreviations: MRI, magnetic resonance imaging; TZ, transition zone; PZ, peripheral zone; NVB, neurovascular bundle.

All patients were followed up for 12 months after treatment. Before and after treatment, the IIEF-5 and sexual domains of the Expanded Prostate Cancer Index Composite (EPIC) were used to assess sexual function at 1, 3, 6, 9, and 12 months post-treatment. Furthermore, sexual function was evaluated according to the differences in patient characteristics and periprocedural data (Figure 1). The target area and treatment range were dependent on the location and size of csPCs in focal therapy. Because the negative influence of HIFU would be hypothesized for erectile function after the treatment of the area close to the NVB, a clinical factor was whether the treatment area included the edge of the PZ with the adjacent NVB. 

### 2.4. Statistical Analyses

Statistical analyses were performed using IBM SPSS^®^ version 26 (IBM, Armonk, NY, USA) and SAS^®^ v9.4 (SAS Institute Inc., Cary, NC, USA). The differences in the characteristics of the patients with and without severe ED were analyzed using the Mann-hitney U test for quantitative data after the normality test (Shapiro–Wilk test). Fisher’s exact test was used for the T stage, Gleason score, and D’Amico risk classification, and the chi-squared test was used for the treatment of the edge of the PZ with an adjacent NVB (treated/untreated). Longitudinal changes in sexual function during the follow-up period were analyzed using one-way analysis of variance (ANOVA). Multivariable logistic regression analysis was used to identify the significant risk factors for ED. Agreement between the predicted and observed risks of severe ED was evaluated using calibration plots. Statistical significance was set at *p* < 0.05.

## 3. Results

### 3.1. Included Patients and Sexual Outcomes after the Treatment

**Figure 2:**
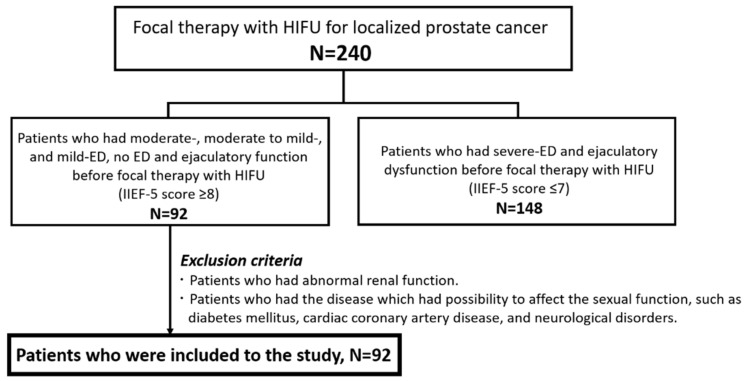
Flow diagram of the patient selection. Abbreviations: HIFU, high-intensity focused ultrasound; IIEF-5, International Index of Erectile Function-5; ED, erectile dysfunction.

Ninety-two patients were included in the study (Figure 2). Of the 240 patients who were treated with focal therapy, the following were included: patients with localized PC who had moderate (IIEF-5 score 8–11, n = 20), moderate-to-mild (IIEF-5 score 12–16, n = 29), mild ED (IIEF-5 score 17–21, n = 34), or no ED (IIEF-5 score 22–25, n = 9) before focal therapy with HIFU without a phosphodiesterase-5 inhibitor or other treatment for ED. The rate of severe ED (IIEF-5 ≤ 7) was 36% 12 months after treatment. In all cases, blood flow disappeared in the treated area on dynamic contrast-enhanced MRI, with an enhanced rim around the treated area. 

### 3.2. Differences in the Patients’ Characteristics and the Peri-Procedural Data according to the Status of Erectile Function after the Treatment

**Figure 3:**
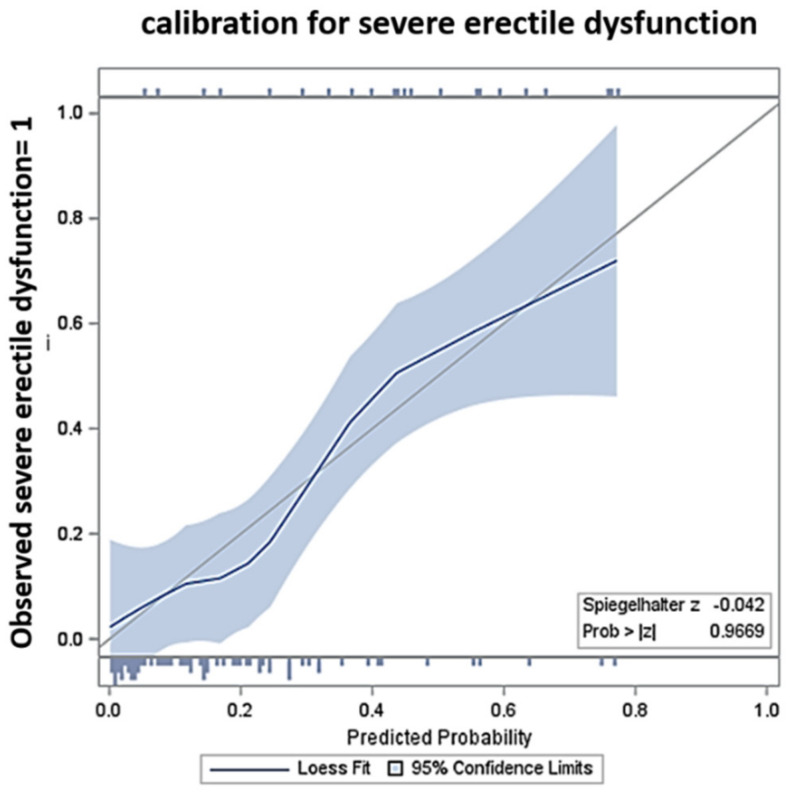
Calibration plots of the fitted model for severe erectile dysfunction after focal therapy with high-intensity focused ultrasound. The gray line indicates the perfect correspondence between the predicted and observed severe ED (perfect calibration), and the blue line shows the calibration of the fitted model. The blue-shaded area indicates the 95% confidence interval.

Table 1 shows the differences in patient characteristics according to the status of erectile function after treatment (moderate, moderate-to-mild, mild, or no ED vs. severe ED). Among patients with and without severe ED after treatment, there were significant differences in the T stage (*p* = 0.044), pre-procedural IIEF-5 score (*p* = 0.002), and pre-procedural score of the sexual domain of EPIC (*p* = 0.011). Table 2 shows the differences in periprocedural data according to the status of erectile function after treatment (moderate, moderate-to-mild, mild, or no ED vs. severe ED). Among the patients with and without severe ED after treatment, there were significant differences in the treatment of the edge of the PZ in proximity to the NVB (treated or untreated, *p* = 0.017). Figure 3 shows the calibration of the model, that is, the agreement between the predicted and observed severe ED after focal therapy with HIFU based on the results from the patients (n = 92). 

### 3.3. Multivariable Logistic Regression Analysis to Predict the Deterioration to Severe Erectile Dysfunction after Focal Therapy with High-Intensity Focused Ultrasound

On multivariable logistic regression analysis, lower pre-procedural scores of IIEF-5 (OR 0.812, *p* = 0.005), lower pre-procedural scores of the sexual domain of EPIC (OR 0.960, *p* = 0.038), and treatment of the edge of the PZ in proximity to NVB (treated vs. untreated) (OR 8.041, *p* = 0.028) were significant risk factors for severe ED after treatment (Table 3). The calibration plots did not show a lack of fit of the fitted multivariable model of the pre-procedural score of IIEF-5, the pre-procedural score of the sexual domain of EPIC, and the treatment of the edge of the PZ in proximity to NVB (treated vs. untreated) to the observed deterioration to severe ED (Figure 3).

### 3.4. Rates of Post-Procedural Condition at 12 Months after Treatment per Pre-Procedural Condition

The rates of post-procedural condition of the patients with no ED, mild ED, moderate-to-mild ED, and moderate ED at 12 months after treatment per pre-procedural condition are shown in Table 4. 

### 3.5. Longitudinal Changes in the International Index of Erectile Function-5 and Expanded Prostate Cancer Index Composite Sexual Domain Scores

**Figure 4:**
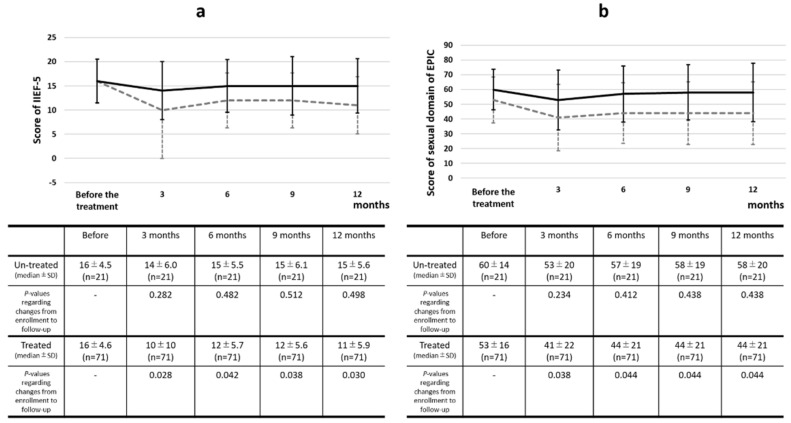
Longitudinal changes in the median score of (**a**) International Index of Erectile Function-5 and (**b**) the sexual domain of the Expanded Prostate Cancer Index Composite with standard deviation. Abbreviations: IIEF-5, International Index of Erectile function-5; EPIC, Expanded Prostate Cancer Index Composite; SD, standard deviation.

Longitudinal changes in the median score of the IIEF-5 and sexual domain scores of the EPIC with standard deviations are shown in Figure 4a,b, respectively. Between patients treated with and without the edge of the PZ in proximity to the NVB, the IIEF-5 and EPIC sexual domains of the EPIC were significantly different at 12 months after treatment. Compared to the pre-procedural scores of IIEF-5 and the sexual domain of the EPIC in patients without treatment of the edge of the PZ in proximity to NVB, the scores declined at 3 months after treatment (*p* = 0.282, *p* = 0.234), and the scores recovered to pre-procedural status at 6 months (*p* = 0.482, *p* = 0.412), 9 months (*p* = 0.512, *p* = 0.438), and 12 months (*p* = 0.498, *p* = 0.438). On the other hand, the scores of IIEF-5 and the sexual domain of the EPIC significantly declined at 3 (*p* = 0.028, *p* = 0.038) and did not recover at 6 (*p* = 0.042, *p* = 0.044), 9 (*p* = 0.038, *p* = 0.044) and 12 months (*p* = 0.030, *p* = 0.044) after treatment in the patients treated with the edge of the PZ in proximity to NVB compared to the pre-procedural scores. 

## 4. Discussion

In the present analysis, a lower pre-procedural score of IIEF-5, a lower preprocedural score of the sexual domain of EPIC, and treatment of the edge of the PZ in proximity to NVB (treated vs. untreated) were significant risk factors for severe ED after the treatment on the multivariable logistic regression analysis. In the longitudinal changes in the scores of IIEF-5 and the sexual domain of the EPIC, the scores of IIEF-5 and the sexual domain of the EPIC were significantly different 12 months after treatment between patients treated with and without the edge of the PZ in proximity to the NVB.

The rates of severe ED after focal therapy with HIFU have been reported to range from 11% to 45% in [6,7,10,11,12]. In this study, severe ED was observed in 24% of the patients at 12 months after treatment, which is similar to previous reports. In multivariable logistic regression analyses, lower pre-procedural scores of IIEF-5, lower pre-procedural scores of the sexual domain of EPIC, and treatment of the edge of the PZ in proximity to NVB (treated vs. untreated) were significant risk factors for severe ED after treatment. A feature of HIFU is heat dissemination to the surrounding cavernous nerve and pudendal arteries and compression of the NVB due to transient prostatic swelling [8], which is caused by diffuse stromal edema of the prostate for a few months immediately after HIFU ablation [13], causing severe ED in up to 75% of men [9]. Furthermore, the treated area appeared as a hypointense zone surrounded by a peripheral rim of enhancement on gadolinium-enhanced T1-weighted images in all patients treated with HIFU [14]. The peripheral rim of enhancement would be considered as inflammation due to the heat effect and was found in the NVB in all patients who were treated at the edge of the PZ in proximity to the NVB in our series. The present results will contribute to the informed consent of patients at risk for severe ED.

In the patients who were treated at the edge of the PZ in proximity to the NVB, the IIEF-5 score and sexual domain of the EPIC declined at 1 month, and the scores increased at 3 months. However, there were significant differences from the pretreatment values for 12 months in the patients in this study. The IIEF-5 score and sexual domain of EPIC declined at 3 months, and there were no significant differences from pretreatment values after 6 months in the patients who were not treated at the edge of the PZ in proximity to the NVB. In a previous longitudinal analysis of erectile function after focal therapy with HIFU, IIEF-5 scores declined at 1 month but improved at 3, 6, 9, and 12 months to pre-procedural conditions [15]. The ED outcomes of post-focal therapy appeared to be independent of these parameters and were influenced only by pre-treatment values [15]. However, in a previous report, the effect of treatment location on erectile function was not evaluated. In this study, the effect of the treatment location was evaluated in patients treated with focal HIFU. Although heat dissemination would cause a permanent heat effect on the NVB, the compression of the NVB owing to transient prostatic swelling would be reduced within a few months. Therefore, it was considered that erectile function continuously deteriorated for 12 months in patients treated at the edge of the PZ in proximity to the NVB because of the heat effect. However, only a transient deterioration of erectile function was observed because of transient compression of the NVB in patients who were not treated at the edge of the PZ in proximity to the NVB. Based on these results, treatment of the edge of the PZ in proximity to the NVB is a significant risk factor for severe ED after focal therapy with HIFU.

In this study, a Gleason score of 4 + 3 or less was considered the main indication for treatment, while a score of 4 + 4 with diameters of 10 mm or less were indicated for treatment based on the patient’s general condition. In a previous review of the consensus of inclusion criteria for focal therapy, almost all studies agreed that focal therapy should be reserved for men with a Gleason score less than 4 + 3; however, selected statements did not reach a consensus in patients with a Gleason score of 4 + 4 [16]. Therefore, the present inclusion criteria were similar to those of other clinical studies on focal therapy, and the present results are expected to be adapted for other clinical studies.

This study had some limitations. First, this was a single-institution study that used pooled data. However, the present results are expected to be adapted for other clinical studies because the present protocol was conducted in accordance with the international consensus. Second, since the number of patients with moderate/moderate-mild/mild/no ED was too small to statistically analyze the effect of each condition, multi-institution studies with a larger number of patients are needed to analyze the functional outcomes in patients treated with focal therapy using HIFU for localized PC. Third, this study was conducted over 12 months; however, longer follow-up periods are warranted to elaborate on the effects of focal therapy in terms of sexual function. Fourth, the relationships between sexual function, mental state, and sexual relations with the partner after treatment were not analyzed in this study. An analysis of this relationship would enhance our knowledge of sexual function after focal therapy. Finally, the relationship between sexual function and serum testosterone levels was not evaluated. The relationship between serum testosterone levels and sexual function after focal therapy will further contribute to the analysis of the mechanisms of sexual dysfunction after treatment. 

## 5. Conclusions

Pre-procedural lower IIEF-5 score, pre-procedural lower score of the sexual domain of EPIC, and treatment of the edge of the PZ in proximity to the NVB were significant risk factors for severe ED after focal therapy. The present results will contribute to the informed consent of patients at risk of severe ED after treatment.

## Figures and Tables

**Table 1:** Differences between the patients’ characteristics and post-procedural erectile function according to the age, PSA value, pre-procedural IIEF-5 score, pre-procedural score of the sexual domain of EPIC using the Mann-Whitney U test in the T stage, Gleason score, and D’Amico risk group using the Fisher’s exact test.

	Patients with Moderate, Moderate-to-Mild, Mild, or No ED (n = 70)	Patients with Severe ED (n = 22)	*p*-Value
**Age (years old), median (range)**	66 (39–78)	67 (52–81)	0.121
**PSA value (ng/mL), median (range)**	6.14 (2.69–16.0)	6.46 (3.17–13.5)	0.492
**Prostate volume (cc), median (range)**	23 (7.0–39)	25 (14–59)	0.625
**T stage (cases)**	T2a: 46	T2a: 8	0.044 *
	T2b: 10	T2b: 7	
	T2c: 16	T2c: 5	
**Gleason score (cases)**	3 + 3: 35	3 + 3: 11	0.669
	3 + 4: 21	3 + 4: 5	
	4 + 3: 5	4 + 3: 1	
	4 + 4: 9	4 + 4: 5	
**D’Amico risk group (cases)**	Low: 26	Low: 7	0.177
	Intermediate: 34	Intermediate: 8	
	High: 10	High: 7	
**Pre-procedural IIEF-5 score**	17 (8–25)	14 (8–19)	0.002 *
**Pre-procedural score of the sexual domain of EPIC**	60 (16–73)	45 (17–65)	0.011 *

* *p* < 0.05. Abbreviations: ED, erectile dysfunction; PSA, prostate-specific antigen; IIEF-5, International Index of Erectile Function-5; EPIC, Expanded Prostate Cancer Index Composite.

**Table 2:** Differences between the peri-procedural data and post-procedural erectile function according to the HIFU ablation time, total HIFU energy, and treated volume using the Mann–Whitney U test in the treatment of the edge of PZ in proximity to NVB using the chi-square test.

	Patients with Moderate, Moderate-to-Mild, Mild, or No ED (n = 70)	Patients with Severe ED (n = 22)	*p*-Value
**HIFU ablation time, minutes, median (range)**	20 (4.0–42)	19 (12–49)	0.424
**Total HIFU energy, K Joule, median (range)**	15,846 (3522–36,824)	16,692 (4828–38,625)	0.320
**Treated volume, cc, median (range)**	11 (2.0–30)	12 (4.0–18)	0.792
**Treatment of the edge of PZ in proximity to NVB (treated/un-treated)**	18/52	3/19	0.017 *

* *p* < 0.05. Abbreviations: ED, erectile dysfunction; HIFU, high-intensity focused ultrasound; PZ, peripheral zone; NVB, neurovascular bundle.

**Table 3:** Multivariable logistic regression analysis to predict the deterioration to severe erectile dysfunction after focal therapy with high-intensity focused ultrasound.

	OR (95% CI)	*p*-Value
**Age**	1.049 (0.973–1.132)	0.214
**T stage (T2a, T2b, vs. T2c)**	2.238 (0.632–7.920)	0.212
**Pre-procedural IIEF-5 score**	0.812 (0.703–0.938)	0.005 *
**Pre-procedural score of the sexual domain of EPIC**	0.960 (0.924–0.998)	0.038 *
**Treatment of the edge of PZ in proximity to NVB (treated vs. untreated)**	8.041 (1.248–51.805)	0.028 *

* *p* < 0.05. Abbreviations: ED, erectile dysfunction; IIEF-5, International Index of Erectile Function-5; EPIC, Expanded Prostate Cancer Index Composite; PZ, peripheral zone; NVB, neurovascular bundle.

**Table 4:** Rates of post-procedural condition at 12 months after the treatment per pre-procedural condition.

		Rates of Post-Procedural Condition at 12 Months after the Treatment Per Pre-Procedural Condition				
		No ED	Mild ED	Moderate-to-Mild ED	Moderate ED	Severe ED
**Pre-procedural condition**	**No ED (n = 9)**	56% (n = 5)	22% (n = 2)	-	11% (n = 1)	-
	**Mild ED (n = 34)**	-	41% (n = 14)	29% (n = 10)	8.8% (n = 3)	21% (n = 7)
	**Moderate-to-mild ED (n = 29)**	-	-	69% (n = 20)	6.9% (n = 2)	24% (n = 7)
	**Moderate ED (n = 20)**	-	-	-	50% (n = 10)	50% (n = 10)

Abbreviations: ED, erectile dysfunction.

## Data Availability

All data are available from the Department of Urology, Tokai University School of Medicine.
